# Neonatal brain oxygenation during thoracoscopic correction of esophageal atresia

**DOI:** 10.1007/s00464-015-4559-1

**Published:** 2015-10-21

**Authors:** Stefaan H. A. J. Tytgat, Maud Y. A. van Herwaarden, Lisanne J. Stolwijk, Kristin Keunen, Manon J. N. L. Benders, Jurgen C. de Graaff, Dan M. J. Milstein, David C. van der Zee, Petra M. A. Lemmers

**Affiliations:** Department of Pediatric Surgery, Wilhelmina Children’s Hospital, University Medical Center Utrecht, KE 04.140.5, P.O. Box 85090, 3508 AB Utrecht, The Netherlands; Department of Neonatology, Wilhelmina Children’s Hospital, University Medical Center Utrecht, KE 04.123.1, P.O. Box 85090, 3508 AB Utrecht, The Netherlands; Department of Anesthesiology, Wilhelmina Children’s Hospital, University Medical Center Utrecht, KE 04.123.1, P.O. Box 85090, 3508 AB Utrecht, The Netherlands; Department of Oral and Maxillofacial Surgery, Academic Medical Center, Meibergdreef 9, 1105 AZ Amsterdam, The Netherlands

**Keywords:** Cerebral oxygenation, Esophageal atresia, Neonate, Thoracoscopy, Near-infrared spectroscopy

## Abstract

**Background:**

Little is known about the effects of carbon dioxide (CO_2_) insufflation on cerebral oxygenation during thoracoscopy in neonates. Near-infrared spectroscopy can measure perioperative brain oxygenation [regional cerebral oxygen saturation (rScO_2_)].

**Aims:**

To evaluate the effects of CO_2_ insufflation on rScO_2_ during thoracoscopic esophageal atresia (EA) repair.

**Methods:**

This is an observational study during thoracoscopic EA repair with 5 mmHg CO_2_ insufflation pressure. Mean arterial blood pressure (MABP), arterial oxygen saturation (SaO_2_), partial pressure of arterial carbon dioxide (paCO_2_), pH, and rScO_2_ were monitored in 15 neonates at seven time points: baseline (T0), after anesthesia induction (T1), after CO_2_-insufflation (T2), before CO_2_-exsufflation (T3), and postoperatively at 6 (T4), 12 (T5), and 24 h (T6).

**Results:**

MABP remained stable. SaO_2_ decreased from T0 to T2 [97 ± 3–90 ± 6 % (*p* < 0.01)]. PaCO_2_ increased from T0 to T2 [41 ± 6–54 ± 15 mmHg (*p* < 0.01)]. pH decreased from T0 to T2 [7.33 ± 0.04–7.25 ± 0.11 (*p* < 0.05)]. All parameters recovered during the surgical course. Mean rScO_2_ was significantly higher at T1 compared to T2 [77 ± 10–73 ± 7 % (*p* < 0.05)]. Mean rScO_2_ levels never dropped below a safety threshold of 55 %.

**Conclusion:**

The impact of neonatal thoracoscopic repair of EA with insufflation of CO_2_ at 5 mmHg was studied. Intrathoracic CO_2_ insufflation caused a reversible decrease in SaO_2_ and pH and an increase in paCO_2_. The rScO_2_ was higher at anesthesia induction but remained stable and within normal limits during and after the CO_2_ pneumothorax, which suggest no hampering of cerebral oxygenation by the thoracoscopic intervention. Future studies will focus on the long-term effects of this surgery on the developing brain.

Congenital esophageal atresia (EA) with tracheoesophageal fistula (TEF) is principally corrected in the neonatal phase. Increasingly, the correction of atresia and closing of the fistula is performed via a thoracoscopic approach [1]. A low-pressure carbon dioxide (CO_2_) pneumothorax (PT) environment collapses the right lung enough to visualize the atresic esophagus and TEF. The procedure is performed under permissive hypercapnic conditions [2, 3]. Recent literature addressing thoracoscopic procedures in neonates has specified some concerns regarding the extra CO_2_ load of applied PT with decreased venous return under elevated intrathoracic pressure. This may be hazardous to neonatal physiology [4] and the developing central nervous system. In particular, impairment of cerebral oxygenation levels in neonates undergoing thoracoscopic procedures has been reported [5].

During surgical procedures and at the patient bedside, neonatal brain hemodynamics and oxygenation can be assessed by transcranial near-infrared spectroscopy (tcNIRS). This tcNIRS permits continuous noninvasive monitoring over extended periods of time. Until now tcNIRS monitoring was not a standard procedure in most neonatal intensive care units (NICU)’s and in OR’s during surgery of newborns. However, in recent years more information has become available concerning the relationship between low cerebral oxygenation monitored by NIRS and the occurrence of brain damage among newborns and young children [6–8]. Currently NIRS monitoring is routinely performed in our NICU and in our OR during thoracoscopic correction of EA. Thus far NIRS findings during thoracoscopic correction of EA of only two patients have been reported [5]. The aim of the present study was to investigate tcNIRS in the perioperative period of neonates elected for minimally invasive EA reconstruction.

## Methods

Guidelines and procedures for this investigation were reviewed and approved by the institutional Medical Ethics Committee of the University Medical Center Utrecht. Parents were informed about the study design and procedures, and informed parental consent was obtained from both parents of each participating neonate. This study was performed in compliance with the principles established in the Helsinki Declaration (version Fortaleza, October 19, 2013). NIRS monitoring is already used as a standard clinical monitoring tool in the NICU of the Wilhelmina Children’s Hospital.

### Patients

In this single-center prospective observational study, 15 patients diagnosed with EA with TEF (Type C atresia), admitted to the NICU of the Wilhelmina Children’s Hospital of the University Medical Center Utrecht were enrolled between January 2012 and September 2014. Preoperative workup consisted of screening for associated anomalies (VACTERL) including ultrasound of the heart and aorta to exclude right descending aortic involvement. Before surgery arterial and venous lines were placed for monitoring blood pressure, arterial blood sampling, and venous access, respectively. A suction drain was placed in the proximal esophageal pouch to prevent aspiration.

### Anesthesia

All patients were subjected to a standardized anesthesia protocol. For the induction of anesthesia sevoflurane (6–8 % inspired concentration) was used with a 40–100 % fraction of inspired oxygen (FiO_2_). After muscle relaxation with atracurium (0.5 mg/kg), the infants were tracheally intubated. Thoracoscopy was performed with both lungs ventilated. Anesthesia was maintained with sufentanil and an oxygen/air mixture and sevoflurane. During the procedure tcNIRS values and simultaneously monitored heart rate, blood pressure, arterial oxygen saturation (SaO_2_), and end tidal CO_2_ values were collected and stored in a high frequency rate (0.5 Hz) on a PC (Bedbase software, UMC Utrecht, NL). Every 30 min blood samples (blood gas; Hb) were taken as part of the routine clinical procedure during thoracoscopic neonatal surgery. The aim was to establish stable anesthetic conditions based on the rScO_2_, SaO_2_ values, end tidal CO_2_ values and blood gas analysis by adjustment of respiratory settings in frequency, maximum inspiratory pressure (Pmax) and FiO_2_. Hypotension was prevented with fluid expansion or inotropes. The CO_2_ gas insufflation was temporarily stopped if the applied PT caused insufficient ability to adequately ventilate the patient.

### Surgery

All surgery was performed in the same operating theater with a stable room temperature of 22 ± 1 °C. In one patient rigid bronchoscopy was performed prior to surgery to assess possible concomitant tracheomalacia and to locate the TEF. The patients were placed in a left laterally recumbent position on a heated operating table (36 ± 1 °C) and tilted 10°–20° reverse Trendelenburg. All thoracoscopic EA repairs were performed through the right thoracic cavity according to earlier described techniques [9]. In short, the PT was created through a 5-mm intercostal camera-trocar placed via an open incisional procedure. An intrathoracic pressure of 5 mmHg was achieved with a flow of 1 L/min insufflation with CO_2_. Two trocars were placed through two 3-mm wounds (one caudodorsal and one cranio-anterior from the camera-trocar). If necessary, an extra trocar was placed to manipulate the lung out of sight to maximize the operating field-of-view. The azygos vein was coagulated and transected when it blocked exposure of the TE junction; the TEF was subsequently ligated with a transfixing absorbable 4.0 Vicryl suture close to the trachea. After transection of the distal esophagus, the proximal pouch was opened. Finally, both ends of the esophagus were anastomosed with interrupted 5.0 absorbable Vicryl sutures over a nasogastric feeding tube.

### Regional cerebral oxygenation

To measure rScO_2_, the INVOS 5100c near-infrared spectrometer (Covidien, Mansfield, Ma USA) was used. The NIRS-determined rScO_2_ was used as an estimator for changes in regional cerebral oxygenation. This measurement provides absolute values, is less sensitive to movement artifacts, and allows for comparison over time. The transducer, containing a light-emitting diode and two distant sensors (i.e., small adult sensor; SAFB-SM, Covidien), was carefully positioned and fixed gently to the frontoparietal surface of the infants head using an elastic band, at the NICU. Differential signals are obtained from these two sensors, and from these signals the rScO_2_ is calculated. The rScO_2_ measures the oxygen saturation of the brain tissue. In a mixture of venous (70–80 %), arterial, and capillary blood the oxygenated hemoglobin (Hb)/total Hb (oxygenated Hb + non-oxygenated Hb) is calculated. Although it still cannot be used as a robust quantitative measurement of cerebral oxygenation, it can serve as a trend monitoring device to detect substantial changes in regional tissue oxygen saturation [10]. The rScO_2_ was monitored during the entire surgical procedure and continued after the patient had returned to the NICU. Detected rScO_2_ levels are considered within safe reference range in (preterm) neonates when values are between 55 and 85 % [11–14]. When rScO_2_ values exceeded reference limits, anesthetic interventions were made according to our NICU protocol as described by Pellicer et al. and Naulaers et al. [15–17].

### Data acquisition

Intraoperative hemodynamic parameters and data on rScO_2_, measured by NIRS (INVOS 4100-5100; Covidien, Mansfield, MA, USA), were continuously monitored and stored for offline analysis using locally developed software (BedBase/SignalBase; University Medical Center Utrecht, Utrecht The Netherlands). Data were analyzed during seven intervals that lasted 10 min. These seven time points were as follows: baseline at the NICU ward (T0), directly after anesthesia induction (T1), 30 min after PT CO_2_-insufflation (T2), 30 min before PT CO_2_-exsufflation (T3), and postoperatively at 6 (T4), 12 (T5), and 24 h (T6).

### Statistical analysis

Data analysis was performed using IBM SPSS statistics software package (IBM^®^ SPSS^®^ Statistics version 20, IBM Corp. Armonk, NY, USA). Data sets are presented as mean ± SD or as median and range when indicated. The data at different time points was analyzed by related parametric or nonparametric methods as appropriate. When no differences were found in an overall analysis across all seven time points, for clinical reasons, analysis subsequently focused on differences between baseline, anesthesia induction and the initial phases of the surgical procedure. Differences between two time points were analyzed with a Student *t* test. Differences between time points with a *p* value <0.05 were considered statistically significant.

## Results

Fifteen patients with type C EA (with distal TEF) had complete data registration and were eligible for analysis. Table 1 presents demographics and clinical characteristics of the patients in this study. Eight patients had associated comorbidities, of which two patients had a right descending aorta and two patients had non-cyanotic cardiac malformations that required surgical correction at a later stage; one patient with dextrocardia and partial anomalous pulmonary venous connection (PAPVC) and one patient with a malaligned ventricular septal defect with overriding aorta. All patients had an overall uneventful thoracoscopic correction of EA. Median time in the OR was 211 [126–387] min, and the median time of PT was 130 [74–260] min.

**Table 1:** Patient characteristics of 15 patients that had a thoracoscopic correction of an esophageal atresia with tracheoesophageal fistula

Clinical characteristics	*N* = 15
Gender (M:F)	10:5
Gestational age (weeks)	39 [36–42]
Postnatal age at surgery (days)	2 [1–7]
Birth weight (grams)	2962 [2155–4490]
Apgar score	
After 1 min	9 [4–10]
After 5 min	9 [5–10]
Other comorbidity	Cor vitium stable hemodynamics 4
No comorbidity *N* = 7	Cor vitium requiring later surgery 2
One or more associated anomalies *N* = 8	Right descending aorta 2
	Tracheomalacia 2
	Duodenal atresia 1
	Anal atresia 1
	Vertebrae/rib deformity 5
Duration (min)	
Time on OR	211 [126–387]
Surgery	148 [83–274]
Pneumothorax	130 [74–260]

Data are presented as median [range]

### Intraoperative hemodynamic and cerebral oxygenation parameters

**Fig. 1:**
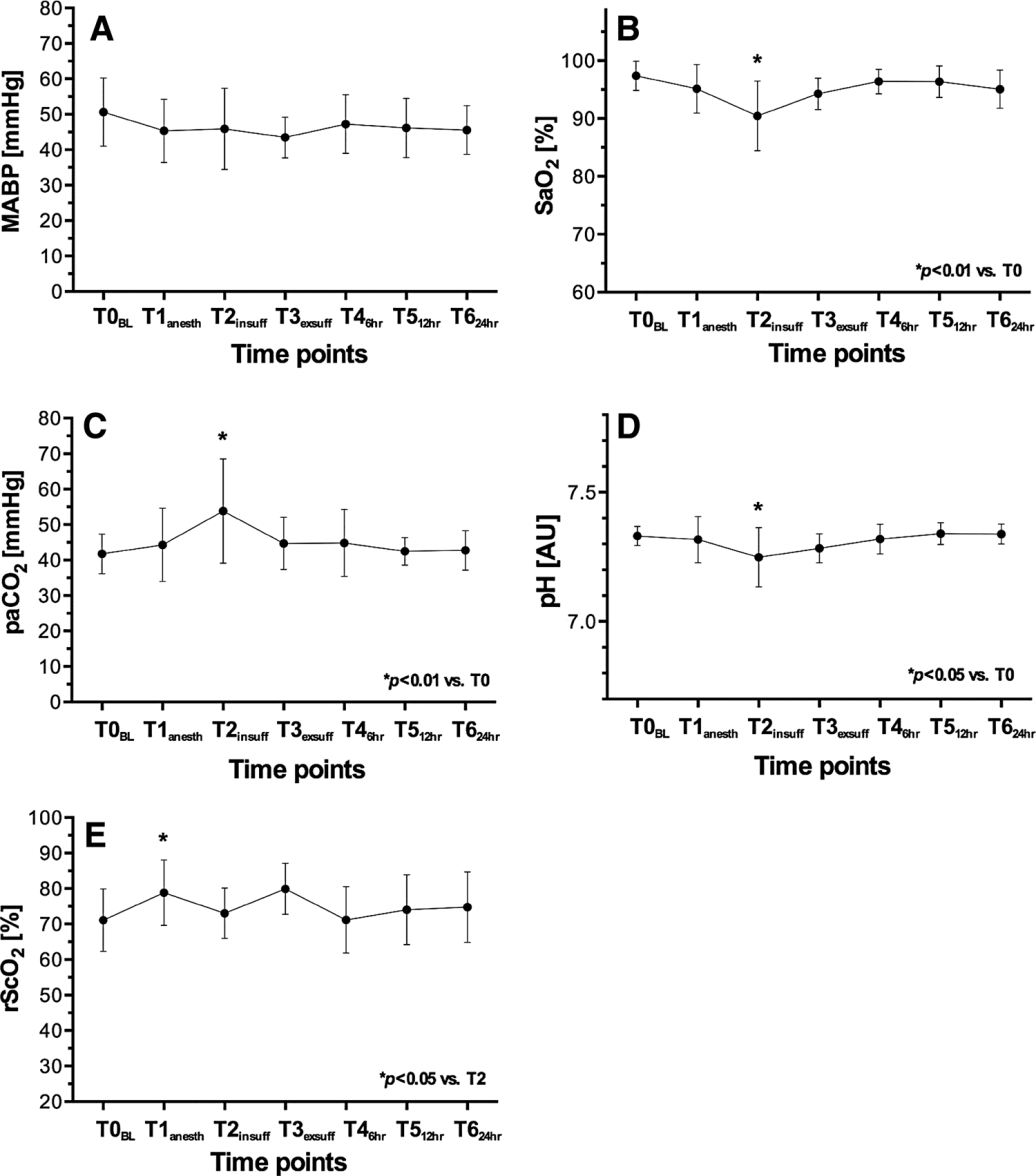
**A**–**E** Perioperative physiological parameters and cerebral oxygenation data at baseline (T0), anesthesia induction (T1), 30 min after CO_2_ insufflation (T2), 30 min before ending CO_2_ insufflation (T3) and in the postoperative phase at 6 (T4), 12 (T5), and 24 h (T6). MABP mean arterial blood pressure (**A**), SaO_2_ saturation of peripheral oxygen (**B**), paCO_2_ partial pressure of arterial carbon dioxide (**C**), arterial sampled pH (**D**), rScO_2_ regional cerebral oxygen saturation (**E**). Significant differences are marked *asterisk* and are presented in the figures with Student *t* test *p* values

Figure 1 summarizes selected perioperative hemodynamic and cerebral oxygenation parameters. All patients remained normothermic during surgery (data not shown). The applied FiO_2_ was 0.68 ± 0.24 at T1 (anesthesia induction), 0.59 ± 0.17 at T2 and 0.49 ± 0.16 at T3 (end of insufflation) (*p* < 0.05). Median inspiratory Pmax [range] was 21 [14–26] cmH_2_O at T2 and 20 [17–28] cmH_2_O at T3. Median respiratory frequency was 42 [30–90] at T2 and 40 [28–69] at T3. Changes in MABP were not significant and measurements remained within physiological range (Fig. 1A). Hypotension was prevented by fluid expansion and administration of dopamine in a range of 1–20 mcg/Kg/min in 10 of 15 (66 %) patients at T2 and to 12 of 15 (80 %) patients at T3. A significant decrease in SaO_2_ was observed at PT application. SaO_2_ decreased from 97 ± 3 % at T0 (baseline) to 90 ± 6 % at T2 (PT application) (*p* < 0.01). SaO_2_ recovered to normal ranges during the procedure (Fig. 1B). PaCO_2_ increased from 41 ± 6 mmHg at T0 to 54 ± 15 mmHg at T2 (*p* < 0.01) and then recovered to normal ranges during the procedure (Fig. 1C). Arterial sampled pH decreased from 7.33 ± 0.04 at T0 to 7.25 ± 0.11 at T2 (*p* < 0.05). It then recovered to normal ranges during the procedure (Fig. 1D). Continuous monitoring of cerebral oxygenation was successful in all 15 neonates. Mean rScO_2_ was significantly higher at T1 compared to T2; 77 ± 10 and 73 ± 7 % respectively (*p* < 0.05). In none of the neonates, the mean rScO_2_ dropped below the safety threshold of 55 % during or after surgery (Fig. 1E).

## Discussion

The aim of this study was to investigate the effects of the installation of a CO_2_ PT on the hemodynamics and cerebral oxygenation of neonates receiving thoracoscopic correction of EA. We observed that surgery with an intrathoracic pressure of 5 mmHg can be performed, while the MABP remains within normal limits. Application of the PT initially lowers SaO_2_, raises paCO_2_, and lowers arterial pH levels. These parameters were corrected through the course of surgery by changes in the ventilation. Under these conditions, cerebral saturation remained within the safety range during the whole procedure. Furthermore, in the postoperative phase cerebral oxygenation and all other monitored parameters remained within normal limits. Our results suggest that a thoracoscopic procedure with a CO_2_ PT set at a pressure of 5 mmHg and flow of 1 L/min can be performed under conditions that allow for reversible arterial blood gas disturbances, with cerebral NIRS values that are elevated during anesthesia induction but that remain stable during and after the PT application.

A large number of pediatric surgical indications can be successfully managed by minimally invasive techniques [18], especially for cases involving treatment of EA. In an increasing number of pediatric surgical centers, these atresias are managed by thoracoscopic interventions [19]. Although benefits of either open or thoracoscopic approach to EA surgery remain to be proven in randomized studies [20], the thoracoscopic approach favors advantages supportive of less postoperative pain, shorter hospital stay [21], less scoliosis caused by rib fusion, decreased respiratory impairment, and improved overall cosmetic result [3, 22, 23].

Despite plausible clinical advantages favoring thoracoscopic EA surgery, concerns have been raised regarding the safety of these thoracoscopic interventions in neonates. These concerns focus mostly on the impact of the applied pressurized CO_2_ PT on neonatal hemodynamics and organ perfusion [3, 4, 20, 24, 25]. Intrathoracic CO_2_ insufflation collapses the lung, which adversely affects the O_2_ and CO_2_ exchange causing hypoxia and hypercarbia. Another factor that causes hypercarbia is the excess systemic CO_2_ load that is absorbed from the thoracic cavity during the PT CO_2_ gas insufflation [5]. Hypoxia and especially hypercarbia with a lowered pH cause vasodilatation of the cerebral vessels in the neonate [26–28]. When lower arterial oxygen saturation causes lower cerebral oxygen supply, the hypercarbia-induced vasodilatation can compensate for the reduced cerebral oxygen supply if blood pressure remains adequate. Diminished perioperative cerebral oxygen saturation during neonatal cardiothoracic surgery is correlated with poor neurodevelopment outcomes [6, 7] and brain magnetic resonance imaging abnormalities at 1 year [8]. Recently, Bishay et al. [5] reported that severe perioperative hypercarbia, acidosis, and decreased cerebral oxygenation were seen in neonates that underwent thoracoscopic surgery for EA and congenital diaphragmatic hernia. It is proclaimed that the reduction in the cerebral oxygenation lingered for up to 24 h postoperatively. However, in the paper by Bishay et al. [5] the rScO_2_ value at the start of operation of 87 % was very high, possibly due to initial hyper-oxygenation. It decreased to 75 % at the end of operation. Also in the postoperative phase, cerebral oxygenation levels remained within reference range.

Furthermore, in the published series on neonatal thoracoscopic interventions [5, 20] that describe the negative impact of CO_2_ application, PT pressures of up to 10 mmHg were applied. A recent experimental study in piglets showed that 10 mmHg PT pressures caused severe hemodynamic instability and decreased cerebral perfusion, whereas these conditions remained stable with PT pressures of 5 mmHg [29]. The results from this piglet study suggest that the applied pressure of 5 mmHg, as used routinely in our clinic, has no severe adverse effects and seems safe to use in thoracoscopic procedures.

In response to the concerns about thoracoscopic EA surgery, Conforti et al. [30] concluded that cerebral oxygenation remains stable during open EA correction. The results of our present study show that this conclusion is not exclusively reserved for open EA correction but that it is also possible during thoracoscopic surgery. We believe that close monitoring and a close collaboration between neonatologists, anesthesiologists, and pediatric surgeons is essential for achieving stable physiological conditions with sustained brain oxygenation levels. According to the anesthesia protocol, fluid expansion and inotropes were applied to prevent blood pressure from declining below physiological limits during the thoracoscopic procedure. Moreover, in the present study we observed that in the initial phase of surgery, installation of the PT caused arterial saturation to drop. To allow adequate ventilation, CO_2_ PT insufflation was then stopped until the patient had recovered. If reinstallation of CO_2_ PT persisted in causing low oxygen saturation, insufflation pressures were not increased but an additional trocar was introduced to gently move the lung away from the operating field-of-view. Initial hypercarbia and consequent acidosis was alleviated during the operation by continuous adjustment of ventilator settings. Acceptable limits of perioperative CO_2_ blood gas values are as yet not known [2, 31]. However, to ascertain CO_2_ and pH levels stayed within acceptable range, adequacy of ventilation is confirmed by arterial blood gas analysis at 30-min intervals [20, 30].

Whether the initial hypercapnia, which can have strong vasoactive effects [2, 26–28] and the acidosis, recorded in our study, could be detrimental for the developing neonatal brain is unknown [31, 32]. These blood gas results are comparable though to those seen during open EA surgery. In our study the initial paCO_2_ during the pneumothorax was 54 mmHg. It was 56 mmHg in a series of patients that were operated via (open) thoracotomy in the study by Bishay et al. [20]. Also, the resulting acidosis in our series with a pH of 7.25 is comparable to a pH of 7.26 in the study of Bishay et al. [20]. In our study, cerebral oxygenation remained stable and not jeopardized during or after the thoracoscopic procedure. The initial increased cerebral NIRS values prior to the surgical procedure are also described in other studies of open or thoracoscopic neonatal surgery [5, 30]. This could be a consequence of an increased supply of oxygen at the phase of anesthesia induction. Also in our study the highest median FiO_2_ was recorded at this time (0.68 ± 0.24).

In conclusion, thoracoscopic EA repair can be performed without the need for conversion or extended procedural times with CO_2_-insufflation pressures that are sustained around 5 mmHg. Cerebral oxygenation was stable within the normal range during and after the procedure. Close perioperative monitoring of neonatal brain oxygenation and close collaboration between surgeons, anesthesiologists and neonatologists will remain part of our operative protocol for patients undergoing thoracoscopic reconstruction of EA. Future studies in our institution will focus on the long-term effects of these types of surgery in neonates and the aim of understanding and avoiding impact on the neonatal brain with adverse neurodevelopmental outcomes.

## Abbreviations

CO_2_: Carbon dioxide
EA: Esophageal atresia
FiO_2_: Fraction of inspired oxygen
Hb: Hemoglobin
MABP: Mean arterial blood pressure
NICU: Neonatal intensive care unit
NIRS: Near-infrared spectroscopy
paCO_2_: Partial pressure of arterial carbon dioxide
Pmax: Maximum inspiratory pressure
PT: Pneumothorax
rScO_2_: Regional cerebral oxygen saturation
SaO_2_: Arterial oxygen saturation
tcNIRS: Transcranial near-infrared spectroscopy
TEF: Tracheoesophageal fistula
